# Optimisation of Maintenance Policies Based on Right-Censored Failure Data Using a Semi-Markovian Approach

**DOI:** 10.3390/s22041432

**Published:** 2022-02-13

**Authors:** Antonio Sánchez-Herguedas, Angel Mena-Nieto, Francisco Rodrigo-Muñoz, Javier Villalba-Díez, Joaquín Ordieres-Meré

**Affiliations:** 1 Department of Industrial Management, School of Engineering, University of Seville, Camino de los Descubrimientos s/n, 41092 Seville, Spain; antoniosh@us.es; 2 Department of Electrical and Thermal Engineering, Design, and Projects, School of Engineering, Campus El Carmen, University of Huelva, 21819 Huelva, Spain; mena@uhu.es; 3 Department of Applied Mathematics II, School of Engineering, University of Seville, Camino de los Descubrimientos s/n, 41092 Seville, Spain; frodrigo@us.es; 4 Hochschule Heilbronn, Fakultät Management und Vertrieb, Campus Schwäbisch Hall, 74523 Schwäbisch Hall, Germany; javier.villalba-diez@hs-heilbronn.de; 5 Escuela Técnica Superior de Ingenieros Industriales (ETSII), Universidad Politécnica de Madrid, 28006 Madrid, Spain

**Keywords:** maintenance interval, maintenance model, semi-Markov process, right-censored data, finite horizon, maintenance cost

## Abstract

This paper exposes the existing problems for optimal industrial preventive maintenance intervals when decisions are made with right-censored data obtained from a network of sensors or other sources. A methodology based on the use of the z transform and a semi-Markovian approach is presented to solve these problems and obtain a much more consistent mathematical solution. This methodology is applied to a real case study of the maintenance of large marine engines of vessels dedicated to coastal surveillance in Spain to illustrate its usefulness. It is shown that the use of right-censored failure data significantly decreases the value of the optimal preventive interval calculated by the model. In addition, that optimal preventive interval increases as we consider older failure data. In sum, applying the proposed methodology, the maintenance manager can modify the preventive maintenance interval, obtaining a noticeable economic improvement. The results obtained are relevant, regardless of the number of data considered, provided that data are available with a duration of at least 75% of the value of the preventive interval.

## 1. Introduction

The main goal of this work is to present a methodology that allows finding the optimal maintenance preventive interval when the failure data are right-censored. In particular, we are interested in finding out how the use of right-censored data affects the calculation of the optimal maintenance interval, considering the temporary maintenance schedule of maintenance interventions and the different types of costs incurred. This study is relevant, since these are the types of data that are generally available in the vast majority of companies, which the maintenance manager must use.

When the maintenance engineer is faced with the problem of managing equipment subject to predetermined preventive maintenance, one of the tasks is to determine the preventive maintenance interval that economically optimises (other factors could also be optimised) the series of interventions that can be carried out on a physical asset over a specified period [1]. For this predetermined maintenance, the interventions can be of two types: corrective interventions originated by a failure of some component of the equipment (failure mode) and preventive interventions carried out after a certain number of hours, kilometres, or units produced. The former is produced randomly, and their number and location in time depend on the failure behaviour of the component with that particular failure mode. The second can be located in time, and the number of can be established along any considered time horizon. In those cases where the failure mode shows wear behaviour, these types of interventions are not independent of each other, since increasing the number of these interventions reduces the number of those due to component failures. However, the increase in the number of the second type of intervention implies an increase in the associated costs of preventive maintenance, but a reduction in the costs of corrective maintenance. Therefore, the optimal preventive maintenance interval that minimises maintenance costs (preventive and corrective) must be found.

To find the optimal interval, the starting point is the existing status and the available information, which must reflect the behaviour in the event of failure, the costs of maintenance interventions, and the income generated by the operation of the equipment [2]. The information on the cost of each intervention is generated during the operation and maintenance phase of the equipment and is usually collected in the Computer-Aided Maintenance Management Systems (CMMS). Once the learning period for maintenance tasks is over, this information is usually relatively stable over time. In the same way, the information on the income from the operation of the equipment is usually collected in the Enterprise Resource Planning systems (ERPs) [3]. Information on behaviour in the event of failure can be obtained from various sources, the equipment manufacturer, the history of failures collected in the plant itself, or any other external database that presents operating conditions similar to that of the equipment under study. The information referring to equipment failures is usually right-censored due to the performance of preventive maintenance. On rare occasions, the maintenance manager has failure data where there is no right-censored data. This information, if it exists, is usually in the hands of manufacturers who have tested their equipment up to its failure point. The maintenance manager cannot bear the test cost, so the adopted policy usually follows the preventive interval set by the manufacturer. Such settings are frequently conditioned by economic interests or brand image. In the research carried out, an extensive data set was available; therefore, the knowledge based impact of right censoring can be identified.

The literature on maintenance is vast, including Reliability Engineering [4,5,6,7,8,9,10,11,12], maintenance policies and modelling [13,14,15,16,17,18,19,20], and optimal preventive maintenance intervals [21,22,23,24]. However, fewer papers address the problem of uncertainty in lifetime distribution [25,26,27,28], either by analysing several time-based maintenance policies having uncertainty in the parameters of the lifetime distribution [29] or using right-censored data [30], and adopt the Markovian approach of transition between states (operational, preventive, and corrective maintenance intervention) [31,32,33,34,35,36].

The discrete-time and continuous-time Markov models satisfy the Markov property, i.e., the future only depends on the present, not on the past. Only the transitions between states and their respective probabilities are considered in the discrete-time model (Markov chain). The sojourn time in each state is irrelevant. In the continuous-time model, the sojourn time is a random variable with an exponential distribution due to the Markovian property. By contrast, in semi-Markov models, the sojourn time in each state does not follow an exponential distribution. It implies that semi-Markov models do not satisfy the Markovian property, which relaxes the constraints and improves the application value [37]. Therefore, successive transitions between states form a Markov chain, called an embedded Markov chain in the semi-Markov model. A wider review, which provides an interesting perspective, can be found in [7,38,39].

Additionally, other authors address the problem of using right-censored data by proposing alternative methods, for example, Li et al. [40] found the life distributions using a histogram-based technique to graphically obtain the maintenance interval. Mazzuchi and van Dorp [26] used the Newton–Raphson method and MLE to try to represent the remaining life of coupler knuckles in railway wagons. Taghipour and Banjevic [41] used the likelihood ratio test to check for trends in the failure data and the ME algorithm to find the parameters for trend analysis applied to pumps located in hospitals.

However, to the best of our knowledge, very few studies provide rules and guidelines to modify the default maintenance interval optimally, and this paper helps to fill that gap. When the maintenance manager has censored data due to the implementation of preventive tasks, the proposed methodology represents an advance with respect to previous works such as [21,22,23,24,42].

This article differs from previous studies in providing the maintenance manager with a prediction of the length of the optimal preventive interval as well as a decision rule to improve the interval being used, based on the analysis of the results of various scenarios, and by using the methodology able to deal with right-censored data. This rule guides the manager in the direction of modification of the preventive interval to be used (increasing or decreasing it) and the intensity of the modification. In this way, the uncertainty induced by using censored data can be overcome. Therefore, to fill the identified gaps, two research questions are formulated:

*RQ1:* How far is the obtained result from the optimal solution when the failure data are censored?

*RQ2:* What strategy would help to move closer to the optimum based on censored data?

The developed methodology guarantees that the solution found is optimal from a mathematical point of view, incorporating the time right-censored data, together with different types of costs and income. In addition, it applies a Semi–Markovian approach of transition between states [43,44,45], plus the *z*-transform, to find the optimal maintenance interval, which, as far as we know, is the first time it has been applied with right-censored data. It is also relevant that the developed methodology enables the assessment and comparison of alternative maintenance policies.

The rest of the paper is organised as follows. Section 2 provides the real data for this study, the research questions formulated, and the methodology applied to develop the mathematical optimisation model based on semi-Markov processes and z-transform. Section 3 presents the results for the different cases studied. In Section 4, those results are highlighted, discussed, and compared. Finally, Section 5 provides the conclusions drawn from the study.

## 2. Materials and Methods

**Figure 1:**
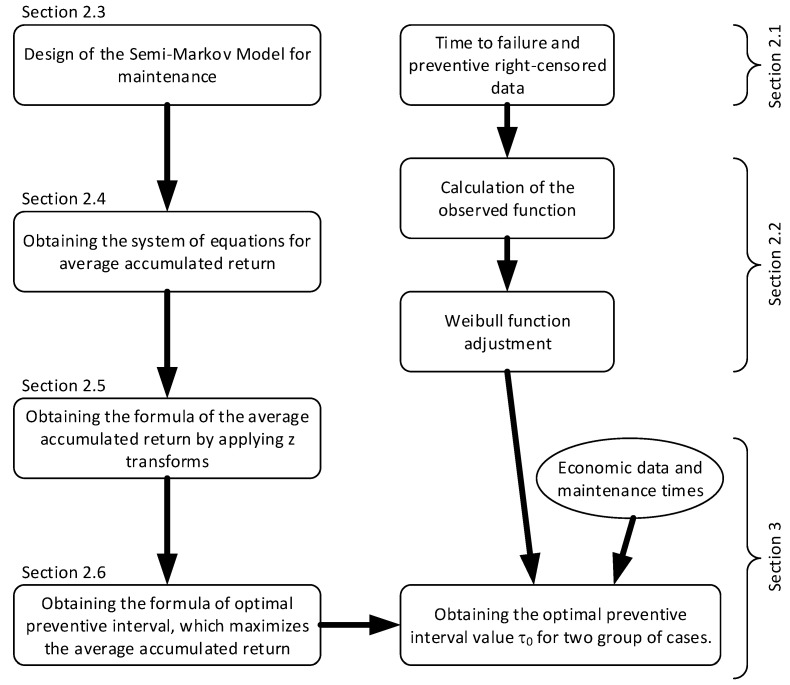
Graphical overview of the process.

To adapt the preventive interval to the particular equipment conditions usage, the manager responsible for maintenance should use the information generated by the equipment itself. The failure information generated is right-censored and it is a challenge to determine the behaviour before the failure of the equipment. The method followed to find the optimal interval when the available failure data is right-censored is explained throughout the article. This process is graphically summarised in Figure 1.

### 2.1. Real Case—Data Selection and Information Processing

This work analyses the behaviour of the O-rings located in the cooled crossover of a 12V diesel engine with 2 litres per cylinder. The crossover is a jacketed tube through which exhaust gases flow. The engine coolant cools this tube, and two O-rings are installed at each end of the tube to prevent coolant leakage. High temperatures affect the O-rings and are responsible for their degradation. In this experiment, failure data have been collected for several years, following the preventive replacement policy of O-rings every 4000 h (scenario A). During this time, it was observed that none of the O-rings had reached this limit, remaining at values very far from this value. This fact allows us to consider that the values of the observed failures can be considered as “not” censored. These failures are listed in Table 1. In addition, in some cases, the O-rings were replaced preventively because the last failure occurred close to the time of the preventive replacement. These are the censored values that are collected in Table 2.

Subsequently, the value of the preventive interval was modified, settling at 1000 h. In this case, the failure data were considerably reduced as many observations were right-censored by the predetermined preventive maintenance performed at 1000 h (scenario B). This censorship radically changed the way of analysing the problem, since the results will depend less on the type of technique or method used and more on the type of censorship [46] and the extent of the censorship. Scenario B, corresponding to the preventive interval of 1000 h, is the one that is usually presented to the maintenance manager.

Failure data and data censored by preventive corresponding to scenario B are, respectively, represented in Table 3 and Table 4. In Table 3, only seven of the nine failures that occurred during the period studied appear, since two of them, those corresponding to 171 h, did not correspond to the failure mode studied.

Table 4 contains 25 censored values and 87 values corresponding to a 1000-h preventive cycle completed.

### 2.2. Determination of the Failure Distribution Function

Data from maintenance interventions are collected at the machine or through an automated procedure using a network of sensors. Its mathematical use for optimisation requires treating these data until obtaining information on the appropriate protocol that can be understood by the model proposed for the simulation. This treatment often supposes a loss of veracity. In other cases, it may happen that the information on which the treatment is started is not adequate. Achieving the most reliable information to the original exposed in the desired protocol is one of the objectives to be achieved in any optimisation process.

In our case, the original starting data could be classified into two groups, those generated during the maintenance activities and those generated during the economic management of those maintenance activities. The first group included failure data and preventive activity data. These data are the hours of operation of the equipment in which preventive and corrective interventions are carried out and the duration of these interventions. In the first group, they were counted from the last repair or preventive change until the failure appeared or until a preventive task was carried out, and the equipment was inactive due to the intervention. The second group of data included the costs of each of the corrective and preventive interventions that were carried out and the income obtained from the use of the equipment.

For the first data group, it was necessary to establish the method to determine the method of mathematically obtaining the failure distribution. Many examples can be found in the literature [47,48]. In our case, a Weibull probability distribution function was used, although other authors analyse different methods to estimate the parameters of the Weibull distribution [49,50]. Other authors have previously used other distributions. However, the Weibull distribution is the one that best adapts to the process of failure appearance in industrial assets. Furthermore, the Weibull distribution includes the exponential distribution and has the advantage of using two or three parameters instead of a single parameter of the exponential function. The exponential survival function has the hazard rate constant, while the Weibull survival distribution extends the exponential distribution to allow constant, increasing, or decreasing hazard rates.

The estimation of the model parameters becomes a difficult task when censorship radically changes the available information and when the opinion of experts does not help to understand the system’s behaviour [51].

Many authors have estimated the parameters of the Weibull distribution for censored data through the Maximum Likelihood Estimation method (MLE), using algorithms for their numerical resolution such as the Maximised Expectation (EM) algorithm, see [52,53,54]. Other authors use and compare various methods such as the Synthetic Minority Over-sampling TEchnique (SMOTE) [55], MLE, Least Square Estimation (LSE) [56], Weibull [57] probability plot, regression of the range of medians with the Benard approximation, and even combine methods, see [58,59]. Bayesian estimators [60] and other types of linear estimators [61] have also been used.

At the same time, the repair and preventive replacement average times and the cost and income average of each type of intervention (corrective or preventive) are also calculated. This last type of data generally poses more difficulties to obtain than to calculate it. However, despite not being as obvious as the calculation of the previous means, the calculation of the failure distribution function can lead to significant management errors, mainly due to the lack of coherence of the starting data. When these failure data are right–censored, due to the performance of preventive maintenance, poor optimisation of the preventive interval will be achieved. This article focuses on the influence of the maintenance management data (first group) to determine the distribution function to perform the preventive interval optimisation calculations.

In this research, a reduced number of data will be used, with which we will obtain an exact solution for the optimal preventive interval. If we had used a large number of data, as is the case with information collected online through sensors, the result would have been the same (exact solution). Nevertheless, in both cases, as will be shown, the results will be far from the optimal solution.

The equipment was failing and was repaired, with few cases of preventive interventions (when the scheduled time according to predetermined maintenance was reached), which also lasted for a few hours of operation. This case represents a situation where the censorship was practically nonexistent, and the observed distribution function was very close to the real one. On the other hand, Table 3 and Table 4 represent the failure and preventive data when a predetermined preventive threshold was established at 1000 h of operation. In this second case, the behaviour of the O-rings would not be known beyond 1000 h. In both cases, it does make sense to estimate the behaviour beyond the predetermined threshold or to analyse the differences between the optimum intervals in both cases. Therefore, a procedure to obtain estimations from the second case closer to those obtained for the first one can be distilled. Looking to gather more information, the following procedure was adopted:

- From the failure data and preventive maintenance data collected in Table 3 and Table 4, the observed function was calculated applying the range of medians method and using the Benard approximation.
- From the observed function, the theoretical Weibull function that fit the best was determined. Then, the least-squares method was used to obtain the theoretical function’s two or three parameters.
- Subsequently, the theoretical function was used to optimise the preventive interval economically.

**Figure 2:**
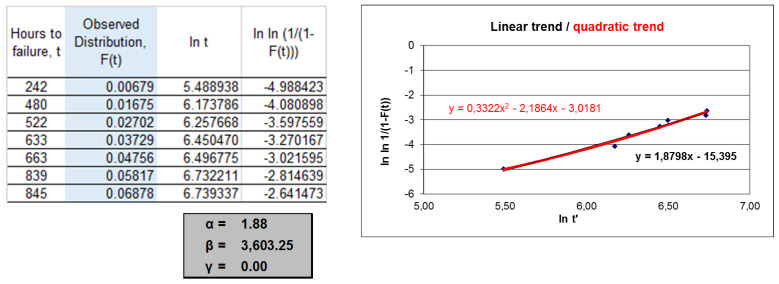
Weibull distribution function obtained from the observed function, which has been determined by Median Rank Regression (MRR) procedure and the Benard approximation.

In Figure 2, the procedures for the data in Table 3 and Table 4 are summarised. The Weibull failure distribution function that best fit the observed function had two parameters, the shape parameter with value 1.88 and a scale parameter (characteristic life) with value 3603. These data were very different from the data obtained for the case of uncensored data and would give rise to a very different preventive interval. The detailed process for the data in Table 1 and Table 2 can be verified in Sánchez-Herguedas et al. [42]. The Weibull functions adjusted to the uncensored data were, in that case, W(2.36, 1317, 0) and W(1.95, 1202, 117), where the first value corresponded to the shape parameter, the second to the scale parameter, and the third to the guaranteed life parameter.

### 2.3. Semi-Markov Maintenance Model with Returns for a Finite Period

To study the behaviour of O-rings concerning failure, we need a mathematical model that reflects this behaviour, such as the semi–Markovian model of three states, which is used with the data that appear in Table 1 and Table 2. This model applies to the predetermined preventive maintenance of much industrial equipment, since it contains the two most representative maintenance states, the state of the equipment when corrective maintenance is performed after a failure (State S2) and the state of the equipment when preventive maintenance is performed after a predetermined number of operating hours (State S3). In these two cases analysed, the data correspond to preventive intervals of 4000 and 1000 h of operation, respectively. State S1 corresponds to the period in which the equipment is performing its required function. In this model, the equipment and others with the same characteristics evolve over time, changing the state according to the law of probability. When the system is in the operational state, the probability of equipment failure is distributed as a Weibull distribution. If the equipment fails, the system goes to the corrective state (S2) and develops a corrective intervention. Suppose the equipment does not fail after a time τ, the system transitions to the preventive state, developing a preventive intervention. Once these maintenance interventions (corrective and preventive) have been carried out, the system returns to its operational state. Both types of interventions have associated costs.

**Figure 3:**
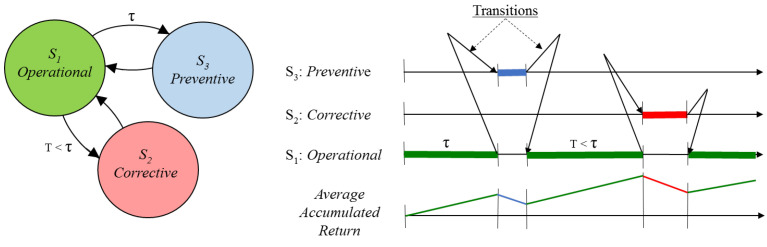
Transition process between states and accumulation of returns.

On the other hand, during the operational state, the equipment generates income. Over time, these costs and income are accumulated in a variable called the average accumulated return, V(m). The optimisation of this variable will allow the calculation of the optimal preventive interval, $\tau_{0}$. These transitions between states and the accumulation of returns are graphically expressed in Figure 3.

### 2.4. Formulating a Difference Equation System for Average Accumulated Return

The model that reflects the behaviour of the system is a semi–Markovian model. The variable to optimise is the average accumulated return in the successive transitions between states. In m transitions, starting from state i, the system accumulates m returns, which added to their respective signs (+ for income and − for costs) will constitute the accumulated return $Q_{i}\left(m\right)$ in *m* transitions from state *i*. For each value of *i* and *m*, the accumulated return is a random variable because once the initial state is determined, the next state is unpredictable, as well as the following states. Thus, in *m* transitions, the system can evolve in many ways. For this reason, it is interesting to know the average accumulated return instead of the accumulated return itself.

Let $v_{i}\left(m\right)=E\left(Q_{i}\left(m\right)\right)$ be the average accumulated return in *m* transitions when the system starts from the initial state *i*. To calculate $v_{i}\left(m\right)$, a difference equation is constructed, separating the *m* transitions into two stages. The first stage is the one constituted by the transition from the initial state *i* to the next state *j*. As the second state can be any of the states of the system, the return $v_{i}\left(1\right)$ in a single transition is a random variable that can reach the values $r_{i1}\left(1\right),r_{i2}\left(1\right),\cdots ,r_{in}\left(1\right)$, with the respective probabilities $p_{i1},p_{i2},\cdots ,p_{in}$. The average return in that transition can be formulated as follows:

(1) $$v_{i}\left(1\right)=\sum_{j=1}^{n}r_{ij}\left(1\right)\cdot p_{ij}.$$

Now, the process continues with the remaining m−1 transitions. Once the first transition has been made, the system is placed in a state called *j*, where *j* takes one of the values 1,2,3,⋯,n. The average accumulated return $v_{j}\left(m-1\right)$ in the following transitions is a random variable that can reach the values $v_{1}\left(m-1\right),v_{2}\left(m-1\right),\cdots ,v_{n}\left(m-1\right)$, with probabilities of $p_{j1},p_{j2},\cdots ,p_{jn}$, respectively, which remain constant throughout the m transitions, since the process is homogeneous. Therefore, the expected value of the return of the remaining m−1 transitions can be formulated as: $v_{j}\left(m-1\right))=\sum_{q=1}^{n}v_{j}\left(m-1\right)\cdot p_{jq}$.

We conclude that the expected average return of the system in m transitions is calculated according to Equation (2):

(2) $$v_{i}\left(m\right)=v_{i}\left(1\right)+v_{j}\left(m-1\right)\cdot p_{j}=v_{i}\left(1\right)+\sum_{q=1}^{n}v_{j}\left(m-1\right)\cdot p_{jq}$$

The vector V(m) is defined as $V\left(m\right)={\left(v_{1}\left(m\right),v_{2}\left(m\right),\cdots ,v_{n}\left(m\right)\right)}^{t}$, where the superscript *t* indicates the transpose in the matrix sense. This last equality can be written as a difference equation:

(3) V(m)=V(1)+P·V(m−1)

where *P* is the matrix of transition probabilities. The Equation (3) gives the average accumulated return in *m* transitions from any possible starting state *i*.

### 2.5. Solving the System of Difference Equations by Applying Transforms Z

Solving this difference equation requires the use of *z* transform and Laurent’s series. To reach its resolution, it is necessary to include the data and functions involved in the calculation: distribution functions of the time spent in each state, the returns for each state, and the matrices that describe the process.

Time-related probabilistic functions for the tree states are as follows:

- F(t) is the Cumulative Distribution Function (CDF) of equipment failures, and *f*(*t*) is the Probability Density Function (PDF). Based on the discussion in Section 2.3, we will use the three-parametric Weibull distribution.
- $G(t_{c})$ is the distribution function of the time the equipment remains under corrective maintenance and $g(t_{c})$ is its probability density function.
- $H(t_{p})$ is the distribution function of the time that the equipment remains under preventive maintenance and $h(t_{p})$ is its probability density function.

Returns (costs for corrective and preventive states and income from operational state) are as follows:

- $R_{1}$, income per time unit that the system remains in State 1 ($S_{1}$: Operational), 6 €/h.
- $R_{12}$, the cost of transition from State 1 to State 2, −4320 €.
- $R_{13}$, the cost of transition from State 1 to State 3, −1 €.
- $R_{2}$, the cost per time unit that the system remains in State 2 ($S_{2}$: Corrective), −95 €/h.
- $R_{21}$, cost of transition from State 2 to State 1, −620 EUR.
- $R_{3}$, the cost per time unit that the system remains in State 3 ($S_{3}$: Preventive), −82 €/h.
- $R_{31}$, the cost of transition from State 3 to State 1, −620 €.

Matrices to describe the process are as follows:

- *P*, the transition probability matrix between states ($p_{ij}$ is the probability of going from State *i* to *j*).
- *F*, stay time matrix ($F_{ij}$ is the average time in State *i* before the system goes to State *j*).
- *R*, returns matrix, where $r_{ij}=R_{i}+R_{ij}$.

With these data, making the *z*-transform of V(m+1)=V(1)+P·V(m), during the calculation, it is required to determine the matrix $I-z^{-1}P$ to reach Equation (4).

(4) $$Z\left[V\left(m\right)\right]=\frac{1}{z-1}V\left(1\right)+{\left(I-z^{-1}P\right)}^{-1}\cdot V\left(1\right)$$

Developing the matrix ${\left(I-z^{-1}P\right)}^{-1}$, the value of Z[V(m)] is reached and decomposed into simple fractions for each of its terms $Z\left[v_{1}\left(m\right)\right],Z\left[v_{2}\left(m\right)\right]$, and $Z[v_{3}\left(m\right)]$. Subsequently, using the appropriate Laurent expansion, the inverse *z*-transform of each one is calculated, obtaining the equations that describe the average accumulated returns in each transition for each of the starting states of the process, $v_{1}\left(m\right),v_{2}\left(m\right)$, and $v_{3}\left(m\right)$. The full development can be followed in Sánchez Herguedas et al. [62]. The average accumulated return in *m* transitions starting in the operating state is described by Equation (5).

(5) $$\begin{array}{cc} v_{1}\left(m\right)= & \frac{1}{4}[\left(2m+1+{\left(-1\right)}^{m-1}\right)\cdot \left(R_{1}\cdot \int_{0}^{\tau }t\cdot f\left(t\right)dt+R_{12}\cdot F\left(\tau \right)+\left(R_{1}\tau +R_{13}\right)\cdot \left(1-F\left(\tau \right)\right)\right)+\\ \; & \left(2m-1-{\left(-1\right)}^{m-1}\right)\cdot (\left(R_{2}\cdot \int_{0}^{\infty }t_{c}\cdot g\left(t_{c}\right)dt_{c}+R_{21}\right)\cdot F\left(\tau \right)+\\ \; & \left(R_{3}\cdot \int_{0}^{\infty }t_{p}\cdot h\left(t_{p}\right)dt_{p}+R_{31}\right)\cdot \left(1-F\left(\tau \right)\right))] \end{array}$$

The average accumulated return in *m* transitions starting in the corrective state is described by Equation (6). To deduce it, the same reasoning is followed as in the previous case.

(6) $$\begin{array}{cc} v_{2}\left(m\right)= & \frac{1}{4}[\left(2m-1-{\left(-1\right)}^{m-1}\right)\cdot \left(R_{1}\cdot \int_{0}^{\tau }t\cdot f\left(t\right)dt+R_{12}\cdot F\left(\tau \right)+\left(R_{1}\cdot \tau +R_{13}\right)\cdot \left(1-F\left(\tau \right)\right)\right)+\\ \; & \left(2m+1+{\left(-1\right)}^{m-1}\right)\cdot \left(R_{2}\cdot \int_{0}^{\infty }t_{c}\cdot g\left(t_{c}\right)dt_{c}+R_{21}\right)+\left(2m-3+{\left(-1\right)}^{m-1}\right)\cdot \\ \; & \left(\left(R_{3}\cdot \int_{0}^{\infty }t_{p}\cdot h\left(t_{p}\right)dt_{p}+R_{31}\right)-\left(R_{2}\cdot \int_{0}^{\infty }t_{c}\cdot g\left(t_{c}\right)dt_{c}+R_{21}\right)\right)\cdot \left(1-F\left(\tau \right)\right)] \end{array}$$

The average accumulated return in *m* transitions starting in the preventive state is described by Equation (7).

(7) $$\begin{array}{cc} v_{3}\left(m\right)= & \frac{1}{4}[\left(2m-1-{\left(-1\right)}^{m-1}\right)\cdot \left(R_{1}\cdot \int_{0}^{\tau }t\cdot f\left(t\right)dt+R_{12}\cdot F\left(\tau \right)+\left(R_{1}\cdot \tau +R_{13}\right)\cdot \left(1-F\left(\tau \right)\right)\right)+\\ \; & \left(2m+1+{\left(-1\right)}^{m-1}\right)\cdot \left(R_{3}\cdot \int_{0}^{\infty }t_{p}\cdot h\left(t_{p}\right)dt_{p}+R_{31}\right)+\\ \; & \left(2m-3+{\left(-1\right)}^{m-1}\right)\cdot \left(\left(R_{2}\cdot \int_{0}^{\infty }t_{c}\cdot g\left(t_{c}\right)dt_{c}+R_{21}\right)-\left(R_{3}\cdot \int_{0}^{\infty }t_{p}\cdot h\left(t_{p}\right)dt_{p}+R_{31}\right)\right)\cdot F\left(\tau \right)] \end{array}$$

### 2.6. Optimisation of the Averaged Accumulated Return When Starting from the Operational State

Deriving and equalising to zero is possible to achieve the mathematical formula of the preventive interval that economically optimises the average accumulated return for each transition, starting from each of the states $\frac{dv_{1}\left(m\right)}{d\tau }=0$, $\frac{dv_{2}\left(m\right)}{d\tau }=0$, and $\frac{dv_{3}\left(m\right)}{d\tau }=0$. The results obtained for each state are:

For the case of starting in the operational state, Equation (8).

(8) $$\tau_{0}^{\alpha -1}=\frac{\beta^{\alpha }}{\alpha }\cdot \frac{-R_{1}}{R_{12}-R_{13}+\frac{\left(2m-1-{\left(-1\right)}^{m-1}\right)}{\left(2m+1+{\left(-1\right)}^{m-1}\right)}\cdot \left(R_{2}\cdot B+R_{21}-R_{3}\cdot C-R_{31}\right)}$$

For cases of starting in the corrective and preventive states, Equation (9).

(9) $$\tau_{0}^{\alpha -1}=\frac{\beta^{\alpha }}{\alpha }\cdot \frac{-R_{1}}{R_{12}-R_{13}+\frac{\left(2m-3+{\left(-1\right)}^{m-1}\right)}{\left(2m-1-{\left(-1\right)}^{m-1}\right)}\cdot \left(R_{2}\cdot B+R_{21}-R_{3}\cdot C-R_{31}\right)}$$

## 3. Results

Applying the procedure followed in Section 2.2 to obtain the Weibull distribution function and Equation (8) to obtain the optimal preventive interval, the results expressed in Table 5 are reached. The average time of corrective and preventive tasks is eight and seven hours, respectively.

Case A corresponded to the uncensored data presented in Table 1 and Table 2. Case B corresponded to the data with censorship, when the maintenance policy implies a preventive change of the O-rings when reaching 1000 h of operation without failure or when the equipment reaches a total of hours that is a multiple of 1000.

In this case, the Weibull function was altered. The failures were spread over a more extended period, and 38% of them occurred after 3600 h. Obviously, this is not what happened when the data were uncensored, and 62% of the failures appeared before reaching 1300 h. The value of the optimal preventive interval given by the model was very high, 10,456. This is due to the flattening and lengthening that the failure distribution curve suffered, as a consequence of the right-censored data. In cases B-2 and B-3, this situation also occurred. Case B-2 considered a failure and the first preventive maintenance at 1000 h. It affected the list of times considered in the Median Rank Regression method. Case B-3 was similar to the previous one; it considered a failure of a preventive maintenance of 1000 h ordered in half of the 87 preventives analysed in Table 4. If the calculations are made only taking into account the failure data and ignoring the censored data at 1000 h, case B-1 is the situation where there is a tendency to group the failures. However, this grouping occurred very early in the life of the O-rings and led to optimising the preventive interval at 353 h. Another group of cases was made up of cases B-4, B-5, and B-6. The distribution of failures was more concentrated, as in case A. However, it was concentrated towards a greater number of hours. For this reason, the optimal preventive interval was established around 2000 h, well above 1059 in case A. All the cases were far from optimal, failing to find a strategy that was close to optimal.

Of course, using this censored data, the optimal preventive interval suitable for a correct economic management of maintenance would not be found. Finding a reason that explains this behaviour is not easy, but it could be thought that the data from A or B cases are not adequate, which could be expected, for example, when two or more failure modes are being mixed.

A further step can be taken in verification. The study can be carried out using the data from case A, Table 1 and Table 2. We can take the data from Table 1 and artificially censor the failures greater than 1000 h. This is how Table 6 is generated. We are in a new scenario C, where we are going to show three different cases. In the case of C-3, only failure data less than 1000 h (42 failures) were considered. The case of C-2 employed failure data up to 1000 h, and censorship corresponding to the cases that failed after more than 1000 h. A total of 41 censored data appeared due to the 41 failures produced over 1000 h. This was intended to generate cases from case A. The data used in the C-3 case were those of the first part of Table 6 (darkened part), and the data used in case C-2 were all the data of Table 6.

Using these data for cases C-2 and C-3, the results shown in Table 7 were obtained. Case C-1 used data from Table 6 and, in addition, from Table 2.

Case C-3 involved an intense concentration of failures around 600 h of operation. The optimal preventive interval was considerably reduced, settling at 418 h. However, case A-2 showed more expected behaviour. There was also a concentration of failures but around 1000 h. The optimal preventive interval increased considerably to 705 h. If cases A-3, A-2, and A-1 are analysed as a whole, they showed a logical progression of the results.

Case D-2 corresponded to a new scenario D, where the data from modified Table 1 were used censoring the data in the range of 400 to 2500 h of operation. Case D-2 corresponded to artificial censorship at 900 h, in the same way that case C-2 corresponded to artificial censorship at 1000 h.

The calculations were carried out for two transitions, starting from the operational state v_1_(2). These cases were compared to obtain the economic differences using Equation (5), which corresponded to the average accumulated return. The results are expressed in Table 8. The fifth column represents the return per hour of operation for each case. It would be the same in all even transitions.

## 4. Discussion

The results in Table 7 show that, when calculating the optimal preventive interval using the formula of our model, its value was always less than the predetermined preventive interval used (censorship). It is observed that, for a preventive interval adopted of 1000 h, case C-1, the derived optimal preventive interval reached the value of 705 h, while, when the preventive interval used was 900 h, case D-1, the optimal preventive interval reached the value of 656 h.

**Figure 4:**
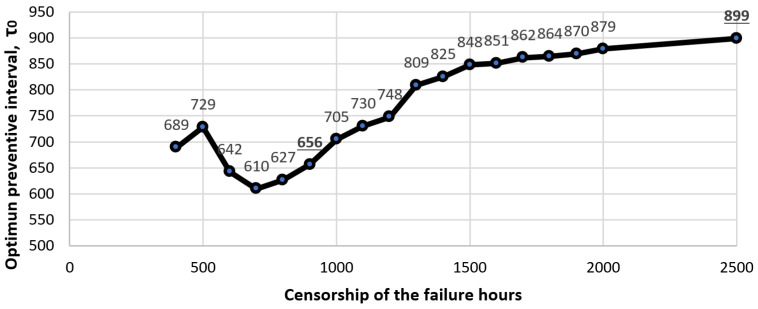
Optimal preventive interval values for case A failures when data are artificially censored.

If with the same data used for cases C-1 and D-1, we modify the value of the predetermined preventive interval used between the range of values 500–2500 h, the behaviour of the optimal preventive interval shows a tendency to increase until reaching the value 899 when the preventive interval used was 2500 h, which was the case for which all fault data were used (see Figure 4). Why would the value 1059 not be reached? This value was not reached because, in these calculations, only the values in Table 2 were considered. This value would have been reached in the calculations if all censored data were considered (Table 2 and Table 6). The increase in the optimal preventive interval between the D-1 case of censorship to 900 h (656) and the case of 2500 h (899) was 27%. This value is comparable to the 22.4% value obtained when verifying the increase in the optimal interval between case A (1059) and case A-0 (822) using the data from Table 2 and Table 6.

In both cases and in other cases with different data, values close to 25% were obtained. This is because the preventive intervals used were 1000 and 900 h, values very close to the optimal 1059 and 899.

**Figure 5:**
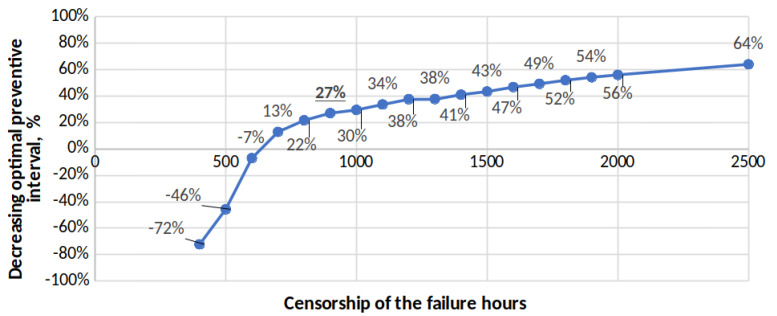
Values of the optimal preventive interval for the failures of case A when the data are artificially censored.

This value close to 25% is of particular interest, because it gives a reference value for the possible variation of the preventive interval when the starting data are censored. We have investigated and obtained a preventive interval of 1059 or 899 h, respectively. However, the maintenance manager does not know if their preventive interval used is close to its optimal value (because they do not let their equipment work until failure). If the value of the optimal interval that the maintenance manager obtains with the model is 30% less than the interval used, the maintenance manager would have a preventive maintenance policy with a high interval, which should be reduced. On the contrary, if the value obtained from the model is 20% less than the interval used, the preventive maintenance would have a low interval, which should be increased. This reduction or increase will be greater or lesser depending on how far the value of the optimal interval obtained from the model was from 25% lower than the interval used, Figure 5. This provides the maintenance manager with a forecast on the length of the optimal preventive interval and a decision rule to improve the interval.

In Figure 4, an abrupt change in the direction of the curve is observed. It can be seen that for values greater than 700 h (point 700 on the abscissa), the curve experiences a change in trend that is maintained until the end. This indicates that the behaviour of the fault distribution function should be studied when the number of failures is very small compared to a large number of censored data due to preventive maintenance. In this case, from values above 75% of the right-censored value (value of the adopted preventive interval), we can find values with which to obtain good to good results.

## 5. Conclusions

To carry out a study where the preventive interval is calculated, it is necessary to have adequate failure data, eliminating from the study those failure data due to other factors (for example, other failure modes). It is usually necessary to have uncensored failure data to optimise the preventive interval, but these are rarely accessible. Most of the data collected by maintenance managers are right-censored due to the performance of predetermined preventive maintenance tasks. The inclusion of censored data in the study introduces uncertainty in calculating the observed function, the theoretical function, the average accumulated return, and the preventive interval. These censored data disturb the calculation of the preventive interval, since they introduce uncertainty. The proposed model allows maintenance managers to use the censored data to guide the modification of the maintenance policy, by optimising the preventive interval, which will have an economic improvement.

The following conclusions can be drawn from using our model’s formula to calculate the preventive interval for right-censored data:

- If the optimal preventive interval obtained using the model is more than 30% lower than the preventive interval used, the maintenance manager can decrease the interval used.
- If the optimal preventive interval obtained is less than 20% less than the preventive interval used, the maintenance manager can increase the interval used.
- This increase or decrease in the value of the optimal preventive interval will be more significant or less depending on how far the value obtained from the model is from 25% lower than the interval used.

This decision rule is of particular importance for the maintenance manager, since it allows them to modify the preventive interval in the correct direction. If censored data are used in the study, the optimal preventive interval increases as we also consider failure data of longer duration (adopt longer preventive intervals). These results are equally relevant regardless of the number of data considered when the number of failures is sufficient to define a failure distribution function according to the behaviour of the physical asset.

This research will continue in the future along two different lines, the first one by using other families of estimators and comparing the results, but the second one intends to extend the model by considering a fourth state for the system. The intention is to split the operational status between regular operational status and degraded operational status, where maintenance would be required by either legal or business constraints forcing consideration of its operation and whether in the new state the probabilistic function for failure becomes different. In this way, it would be possible to enable a stronger alignment between production and maintenance policies, where a more integrated perspective for the business as a whole is envisaged.

## Figures and Tables

**Table 1:** Hours of operation of the O-rings until failure in Scenario A.

Operation Hours to Failure											
190	276	296	409	429	430	437	454	481	492	498	499
543	552	552	552	552	577	603	604	604	612	619	658
675	683	696	702	742	754	773	797	812	836	881	889
912	913	942	974	994	994	1014	1015	1024	1025	1041	1105
1183	1203	1211	1236	1238	1240	1249	1274	1295	1304	1312	1343
1345	1407	1413	1421	1442	1447	1486	1492	1542	1581	1601	1621
1630	1675	1735	1892	1926	1960	2006	2101	2242	2437	2450	

**Table 2:** Hours of operation of the O-rings with censorship due to preventive maintenance policy in scenario A.

Operation Hours to the Censorship											
45	84	84	103	121	176	199	217	259	324	371	387
428	508	514	519	638	655	661	735	760	764	766	769
790	867	986	1006	1011	1111	1167	1188	1188	1395	1396	1505
1752	2016										

**Table 3:** Hours of operation of the O-rings until failure in Scenario B.

Operation Hours to Failure						
242	480	522	633	663	839	845

**Table 4:** Hours of operation of the O-rings with censorship due to preventive maintenance policy in scenario B.

Operation Hours to the Censorship											
93	93	128	128	128	128	155	161	220	220	240	240
337	367	380	380	467	467	478	485	485	520	758	829
829	1000	1000	1000	1000	1000	1000	1000	1000	1000	1000	1000
1000	1000	1000	1000	1000	1000	1000	1000	1000	1000	1000	1000
1000	1000	1000	1000	1000	1000	1000	1000	1000	1000	1000	1000
1000	1000	1000	1000	1000	1000	1000	1000	1000	1000	1000	1000
1000	1000	1000	1000	1000	1000	1000	1000	1000	1000	1000	1000
1000	1000	1000	1000	1000	1000	1000	1000	1000	1000	1000	1000
1000	1000	1000	1000	1000	1000	1000	1000	1000	1000	1000	1000
1000	1000	1000	1000	1000	1000	1000	1000	1000	1000		

**Table 5:** Results of the cases (cases A, B, B-1, B-2, B-3, B-4, B-5, B-6) analysed. Parameters of the Weibull distribution function and the optimal preventive interval.

	Weibull			Optimal Preventive Interval $\tau_{0}$
	Shape Parameter α	Scale Parameter β	Guaranteed Life γ	
Case A (uncensored)	2.36	1317	0	1059
Case B	1.88	3603	0	10,456
Case B-1	2.42	695	0	353
Case B-2	1.85	3729	0	11,914
Case B-3	1.90	3541	0	9805
Case B-4	2.65	1939	0	1908
Case B-5	2.40	2265	0	2663
Case B-6	2.48	2150	0	2369

**Table 6:** Failure hours of the O-rings in the case A, adapted to a policy of preventive replacement every 1000 h.

Time to Failure (Hours) and Censored Data (1000 h)					
190	276	296	409	429	430
437	454	481	492	498	499
543	552	552	552	552	577
603	604	604	612	619	658
675	683	696	702	742	754
773	797	812	836	881	889
912	913	942	974	994	994
1000	1000	1000	1000	1000	1000
1000	1000	1000	1000	1000	1000
1000	1000	1000	1000	1000	1000
1000	1000	1000	1000	1000	1000
1000	1000	1000	1000	1000	1000
1000	1000	1000	1000	1000	1000
1000	1000	1000	1000	1000	

**Table 7:** Results of the cases (cases A, C-1, C-2, C-3, and D-2) analysed. Parameters of the Weibull distribution function and the optimal preventive interval.

	Weibull			Optimal Preventive Interval $\tau_{0}$
	Shape Parameter α	Scale Parameter β	Guaranteed Life γ	
Case A (uncensored)	2.36	1317	0	1059
Case C-1 (1000 + censored)	2.79	1149	0	822
Case C-2 (1000 + censored)	3.34	715	0	418
Case C-3 (1000 + censored)	2.76	1042	0	705
Case D-2 (900 + censored)	2.94	991	0	656

**Table 8:** Economic comparison of cases A, C-1, C-2, C-3, and D-2.

Cases	Average Return of Two Transitions v_1_(2) (€)	Average Transition (h)	Number of Transitions	Average Return (€/h)
Case A (uncensored)	2174.36	899.5	2	1.209
Case C-1 (1000 + censored)	1815.24	745.6	2	1.217
Case C-2 (1000 + censored)	1388.05	647.0	2	1.073
Case C-3 (1000 + censored)	530.29	402.7	2	0.658
Case D-2 (900 + censored)	1308.91	610.4	2	1.072

## Data Availability

Data sample can be obtained from the authors upon request.

## Abbreviations

The following abbreviations are used in this manuscript:

CDF	Cumulative Distribution Function
CMMS	Computer-Aided Maintenance Management Systems
ERPs	Enterprise Resource Planning systems
LSE	Least Square Estimation
ME	Maximised Expectation algorithm
MLE	Maximum Likelihood Estimation
MRR	Median Rank Regression
PDF	Probability Density Function
SMOTE	Synthetic Minority Oversampling Technique
